# Genotyping of the Pseudorabies Virus by Multiplex PCR Followed by Restriction Enzyme Analysis

**DOI:** 10.5402/2011/458294

**Published:** 2011-11-24

**Authors:** Antônio Augusto Fonseca, Cristina Gonçalves Magalhães, Érica Bravo Sales, Régia Maria D'Ambros, Janice Ciacci-Zanella, Marcos Bryan Heinemann, Rômulo Cerqueira Leite, Jenner Karlisson Pimenta dos Reis

**Affiliations:** ^1^LANAGRO/MG, Avenida Rômulo Joviano, Caixa Postal 50, Centro, 33600-000 Pedro Leopoldo, Minas Gerais, CEP, Brazil; ^2^CEDISA, Concórdia, Santa Catarina, Brazil; ^3^Embrapa Suínos e Aves, Concórdia, Santa Catarina, Brazil; ^4^Escola de Veterinária, Universidade Federal de Minas Gerais, Belo Horizonte, Minas Gerais, Brazil

## Abstract

Suid herpesvirus 1 (SuHV-1) is the causative agent of Aujeszky's disease. The infectious agent has only one serotype, but it was classified by restriction enzyme analysis of the whole genome into four genotypes, named I to IV. The aim of this study was to standardize a rapid method for genotyping SuHV-1 without virus isolation, using a multiplex-PCR followed by enzymatic restriction analysis. The complete genome of the virus was analyzed *in silico* to determine the restriction sites for the enzyme *BamH*I. Primers were designed to flank sites with emphasis on certain points of differentiation of genotypes. The standard PCRs were able to detect the SuHV-1 and also to differentiate genotypes from brain tissue of infected pigs. The *BamH*I-PCR is a rapid, practical, and sensitive way to genotype SuHV-1.

## 1. Introduction

Suid herpesvirus 1 (SuHV-1) is the causative agent of Aujeszky's disease, a disease of great impact on the swine industry. Due to the important economic losses caused by this disease, notification of any outbreak is mandatory [1].

SuHV-1 belongs to the family Herpesviridae, subfamily Alphaherpesvirinae, genus *Varicelovirus* [2]. The main host of SuHV-1 is the pig. Infected young pigs suffer from neurological damage, whereas infected adults suffer from abortion and respiratory signals. SuHV-1 has only one serotype, and virus differentiation is made by molecular techniques, in particular by the restriction fragment length pattern (RFLP), allowing the differentiation of circulating viral strains. The genotyping of samples by RFLP was fundamental to understand the epidemiology of SuHV-1 [3]. Despite the development of other molecular typing techniques, the main method of characterizing the SuHV-1 is still digesting the full genome by the enzyme *BamH*I (*BamH*I-RFLP) [4–8]. This methodology was used by several authors to characterize SuHV-1 isolates in Europe, Japan, Argentina, and Brazil [7–16].

The *BamH*I-RFLP is able to discriminate the SuHV-1 into four genotypes. Genotypes I and II are distributed worldwide, and genotypes III and IV, originally described in Denmark and Thailand, respectively, were no longer reported [3]. Currently, genotype I is prevalent in populations of wild boars in Europe [8].

The disadvantages of *BamH*I-RFLP are the need for virus isolation and a time-consuming electrophoresis. Some variations of the methodology require equipments such as ultracentrifuges for viral DNA purification [7]. The small amount of viral DNA and the lack of facilities for virus isolation are also critical in the implementation of this technique [8].

The aim of this work was to develop a fast multiplex PCR followed by enzymatic restriction analysis using *BamH*I enzyme that allows the characterization of SuHV-1 without the need of virus isolation, even in samples with small viral DNA amounts.

## 2. Material and Methods

### 2.1. Primer Design

Primers were designed based on *BamH*I restriction sites found in the complete genome of SuHV-1 available in GenBank (BK001744) [17], using the software WebCutter (http://rna.lundberg.gu.se/cutter2/). The restriction sites were compared with the restriction maps previously described [3]. After detection of the best regions, the sequences were submitted to the program Pimer3Plus [18]. The amplicon sizes were chosen according to the restriction fragments that would be generated after enzymatic digestion and the possibility of their use in a multiplex PCR (Tables 1 and 2).

### 2.2. Samples

Eleven isolates of SuHV-1 (five of genotype I and six of genotype II), previously characterized in other works [16, 19], the vaccine strain Bartha, standard sample Shope, and attenuated isolate NIA-4, were used for PCR development. All samples were propagated in PK15 cells before DNA extraction. 

Twenty clinical brain samples of pigs positive for SuHV-1 by virus neutralization and virus isolation, collected from outbreaks in the state of Santa Catarina, Brazil, in the years of 2002 and 2003 were used to standardize the technique in tissue sample. Ten negative samples of the same tissue were also used to determine whether the PCRs would be able to detect the virus without unspecific reactions. All samples were previously characterized in other works [20]. 

Total DNA was extracted by commercial kit Wizard Genomic DNA Purification (Promega, USA) and stored at −20°C for later genotyping.

### 2.3. Multiplex PCR (mPCR)

The reactions were developed by testing different parameters such as annealing temperature, concentration of the reagents (primers, DNA polymerase, MgCl_2_), and duration of each stage. The first tests were performed with separate primers for subsequent use in the multiplex. 

 The multiplex PCRs were standardized using two sets of primers: mix A, which contained *BamH*I-1-738, *BamH*I-3-256, and *BamH*I-6/7-637, and mix B, which contained primers *BamH*I-4-357, *BamH*I-19/20-519, and *BamH*I-16/17-799, Both of the mixes generate three bands prior to restriction enzyme. A 20 *μ*L reaction was standardized with the following concentrations of reagents: 10 pmol of each primer, 4 *μ*L of 5x buffer GoTaq Green (Promega, USA), 2.0 mmol/L magnesium chloride, 6% DMSO, 300 mmol/L DNTP (Invitrogen, USA), and 3 U GoTaq HotStart (Promega, USA). The conditions used for both PCR were as follows: a denaturing stage at 95°C for 5 min., 35 cycles at 95°C for 50 s, 60°C for 50 s, 72°C for 1 min., and a final stage extension at 72°C for 7 min.. Positive and negative controls were used in all the PCR mixes to check for contamination and unspecific amplifications. 

Enzymatic restriction of the mPCR with *BamH*I was performed according to the manufacturer's instructions (New England Biolabs, USA). The gels were analyzed on 2.5% agarose gel stained with ethidium bromide at 0.5 mg mL^−1^ after one hour of running.

## 3. Results

### 3.1. Primer Design

The primers chosen for PCR target nine restriction sites for *BamH*I detected in the *in silico* analysis of complete genome (Table 1). The regions were chosen because they are the key points used to differentiate genomic types. Among the chosen primer pairs, *BamH*I-1-738 and *BamH*I-4-357 are most important for the differentiation of all genotypes. Genotype II has no restriction site in *BamH*I-1-738, and genotype III has no restriction site in *BamH*I-4-357 (Table 2). These same areas offer the opportunity to subtyping with the enzyme BstEII, which has a restriction site in *BamH*I-4-357 for genotype I. The other primers were selected because the regions can be used to differentiate some SuHV-1 strains, like the standard strain Shope, used in our laboratory for virus neutralization tests.

### 3.2. mPCR

**Figure 1:**
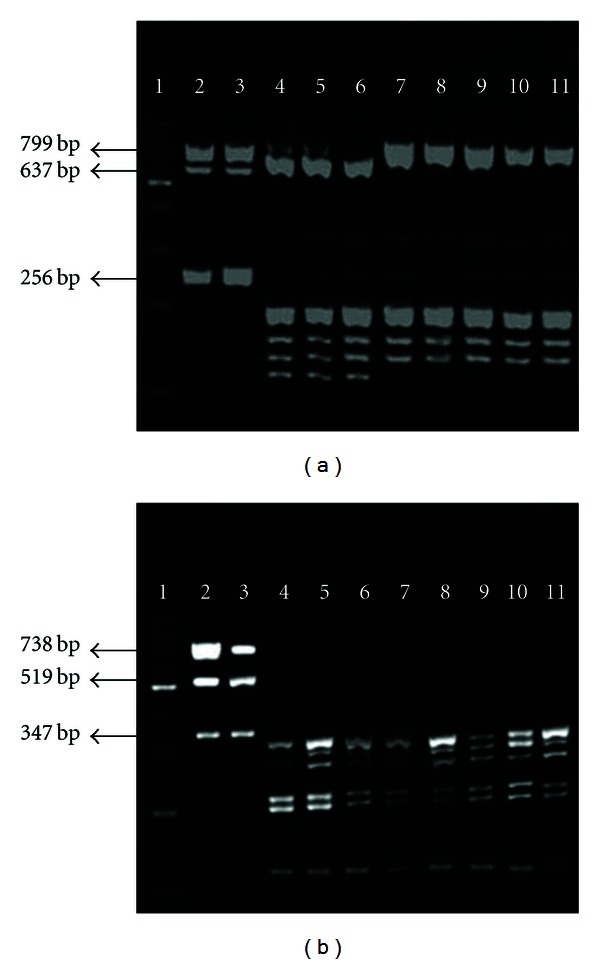
Profiles of enzymatic restriction by *BamH*I for isolates and clinical samples. (a): mix A and (b): mix B. The samples were selected as examples. Genotypes I (lanes 4 to 6) and II (lanes 7 to 11) were differentiated using mix A. Lanes: (1) molecular size marker, (2) sample Shope (without enzymatic restriction), (3) clinical sample (without enzymatic restriction), (4) standard sample Shope, (5) isolate 936, (6) isolate NIA-4, (7) isolate 3319, (8) isolate 3370, and (9)–(11) clinical samples.

The *BamH*I-mPCR was first developed with a set of six primer pairs in one reaction, but good visualization of the results could only be possible after two hours of electrophoresis. The use of two mixes allows a cleaner visualization of the restriction enzyme profiles. It was possible to differentiate genotypes I and II using enzymatic restriction after DNA amplification with mix A (Figure 1(a)). Mix B was used to differentiate specific strains like standard strain Shope (Figure 1(b)).

### 3.3. Samples

Among the twenty clinical samples, seventeen amplified as expected. Three samples required the addition of a larger amount of DNA for better visualization of bands in agarose gel. The clinical samples negative for SuHV-1 showed no bands, indicating the reaction specificity. All isolates previously characterized in other studies [15, 16] showed the expected restriction patterns for genotype II (Figure 1).

## 4. Discussion

The *BamH*I-RFLP is one of the most used methods for molecular characterization of SuHV-1. Although sequencing methods are considered more accurate [8, 18], the restriction enzyme analysis is still important for genotyping of SuHV-1. The biggest problems of RFLP are the need of ultracentrifuges, the necessity of virus isolation and the electrophoresis that takes over eight hours under controlled temperature [14, 16]. The multiplex PCR followed by restriction with *BamH*I (*BamH*I-mPCR) developed in this study allows the analysis of restriction sites for genotyping of SuHV-1 directly from clinical samples without the need of any equipment other than that of a basic molecular biology laboratory. 

The *BamH*I-mPCR was able to detect the virus in all the clinical samples, which demonstrated its importance for genotyping SuHV-1 when there is a shortage of equipment or isolation is not possible. We have previously developed a nested PCR for the same purpose in our laboratory [20], but it is a more laborious and time-consuming technique and very prone to contamination. 

PCR followed by restriction enzyme has been used characterize other viruses of the Herpesviridae family, such as bovine herpesvirus 1, 2, and 5 [21], herpes simplex 1 [22, 23], varicella-zoster virus, and human cytomegalovirus [24]. These techniques are simple and easy to perform in molecular biology laboratories and are very useful in epidemiological studies. Genotyping of SuHV-1 allows a better understanding of its epidemiology and helps to weigh the impacts of this infectious agent and, ultimately, to reduce or even avoid the losses it produces. Molecular techniques have been used with many objectives such as to verify the introduction of new genotypes in a country [10], to characterize field isolates derived from vaccines samples [11] and even to identify SuHV-1 isolates from wild animals, such as the wild boar [8]. The *BamH*I-mPCR is a fast and useful method for genotyping that allows for comparison with results from the published data of the last twenty-five years since the beginning of the use of *BamH*I-RFLP technique.

## Figures and Tables

**Table 1:** Primers used in this work.

Primer	Sequence (5′–3′)	Position*	Amplicon	Gene
*BamH*I-1-738-F	TACCAGATCGGTTGATGTGC	1227–1246	738	*UL56*
*BamH*I-1-738-R	AACAGGAGCGTCTCCGAGTA	1945–1964		
*BamH*I-3-256-F	GTAGGCCGCGTAGAACTGC	37671–37689	256	*UL36*
*BamH*I-3-256-R	GCGCATCGAGAGCAAGTA	37909–37926		
*BamH*I-4-357-F	GAGCAGCATGATCGTCGTC	55372–55390	357	*UL44, UL26, UL26.5*
*BamH*I-4-357-R	TTCGTCTCGCAGATGATGTC	55709–55728		
*BamH*I-6/7-637-F	GAGTCCAAGGACATGGAGGA	62380–62399	637	*UL22*
*BamH*I-6/7-637-R	GTCTCACACACAACCGGGTA	62997–63016		
*BamH*I-16/17-799-F	GAGATGCACCTGATCGACCT	119085–119104	799	*US3, US4*
*BamH*I-16/17-799-R	AAGACGAGCACGACGATGTA	119883–119864		
*BamH*I-19/20-519-F	CGCCGTTCTACATCACCAC	126073–126091	519	*US2*
*BamH*I-19/20-519-R	ATCCTGCCGTCTAGGAGATG	126572–126591		

^∗^Complete SuHV-1 genome (GenBank access: BK001744).

**Table 2:** Number of restriction sites in each amplicon in SuHV-1 genotypes I, II, and III.

	Restriction sites in each genotype		
Primer	I	II	III
*BamH*I-1-738-F	1	0	1
*BamH*I-1-738-R			
*BamH*I-3-256-F	1	1	1
*BamH*I-3-256-R			
*BamH*I-4-357-F	1	1	0
*BamH*I-4-357-R			
*BamH*I-6/7-637-F	2	2	2
*BamH*I-6/7-637-R			
*BamH*I-16/17-799-F	2	2	2
*BamH*I-16/17-799-R			
*BamH*I-19/20-519-F * BamH*I-19/20-519-R	2	2	2
